# Accumulation of tissue-resident natural killer cells, innate lymphoid cells, and CD8^+^ T cells towards the center of human lung tumors

**DOI:** 10.1080/2162402X.2023.2233402

**Published:** 2023-07-11

**Authors:** Demi Brownlie, Andreas von Kries, Giampiero Valenzano, Nicole Wild, Emel Yilmaz, Jesper Säfholm, Mamdoh Al-Ameri, Evren Alici, Hans-Gustaf Ljunggren, Igor Schliemann, Ozan Aricak, Felix Haglund de Flon, Jakob Michaëlsson, Nicole Marquardt

**Affiliations:** a Center for Infectious Medicine, Department of Medicine Huddinge, Karolinska Institutet, Huddinge, Sweden; b Center for Hematology and Regenerative Medicine, Department of Medicine Huddinge, Karolinska Institutet, Huddinge, Sweden; c Institute of Environmental Medicine, Karolinska Institutet, Stockholm, Sweden; d Department of Molecular Medicine and Surgery, Karolinska Institutet, Karolinska University Hospital, Stockholm, Sweden; e Haematology Centre, Karolinska University Hospital, Huddinge, Sweden; f Department of Clinical Pathology and Cytology, Karolinska University Hospital, Stockholm, Sweden; g Department of Oncology-Pathology, Karolinska Institutet, Stockholm, Sweden; h Department of Laboratory Medicine, Division of Pathology, Karolinska Institutet, Huddinge, Sweden

**Keywords:** CD8+ T cells, ILC, lung cancer, NK cells, tissue-resident, tumor-infiltrating

## Abstract

Lung cancer is a leading cause of cancer-related death worldwide. Despite recent advances in tissue immunology, little is known about the spatial distribution of tissue-resident lymphocyte subsets in lung tumors. Using high-parameter flow cytometry, we identified an accumulation of tissue-resident lymphocytes including tissue-resident NK (trNK) cells and CD8^+^ tissue-resident memory T (T_RM_) cells toward the center of human non-small cell lung carcinomas (NSCLC). Chemokine receptor expression patterns indicated different modes of tumor-infiltration and/or residency between trNK cells and CD8^+^ T_RM_ cells. In contrast to CD8^+^ T_RM_ cells, trNK cells and ILCs generally expressed low levels of immune checkpoint receptors independent of location in the tumor. Additionally, granzyme expression in trNK cells and CD8^+^ T_RM_ cells was highest in the tumor center, and intratumoral CD49a^+^CD16^−^ NK cells were functional and responded stronger to target cell stimulation than their CD49a^−^ counterparts, indicating functional relevance of trNK cells in lung tumors.

In summary, the present spatial mapping of lymphocyte subsets in human NSCLC provides novel insights into the composition and functionality of tissue-resident immune cells, suggesting a role for trNK cells and CD8^+^ T_RM_ cells in lung tumors and their potential relevance for future therapeutic approaches.

## Introduction

Lung cancer is one of the most common causes of cancer-related death. Current immunotherapeutic approaches mainly focus on circulating T cells which infiltrate the tumor. However, little is known about the distribution of tissue-resident lymphocyte subsets such as tissue-resident NK (trNK) cells, innate lymphoid cells (ILCs), and tissue-resident memory T (T_RM_) cells within lung tumors.

TrNK and T_RM_ cells, defined by co-expression of CD69, CD49a, and CD103, are readily identified in human lung^1,2^. ILCs which express high levels of CD69 are considered tissue-resident irrespective of CD49a or CD103 expression^2–4^. TrNK cells are characterized by a CD16^−^ phenotype but differ markedly from conventional circulating CD16^−^ NK cells both at the protein and transcriptome levels^2^. Frequencies of CD16^−^ NK cells are increased in lung tumors^5–7^, suggesting that tumor-infiltrating NK cells are, at least in part, comprised of trNK cells. Additionally, a fraction of NK cells in human lung tumors have been reported to express immune checkpoint receptors, including PD-1, CTLA-4, and TIM-3^8,9^, potentially inhibiting cytotoxic function of intratumoral NK cells.

In addition to NK cells and T_RM_ cells, ILC1, ILC2, and ILC3 have been identified in human lung tumors in several studies^10,11^. While ILC2s are reduced in frequency in lung tumor tissue compared to tumor-free lung tissue, NKp44^+^ ILC3s have been suggested to aid the formation of intratumoral lymphoid structures, indicating a relevance for ILC subsets within lung tumors^10^.

Compared to trNK cells and ILCs, CD8^+^ T_RM_ cells have been studied in more detail in lung cancer. CD8^+^ T_RM_ cells are more frequent in lung tumors compared to peritumoral lung tissue^12^, and their frequencies are associated with greater overall survival^13,14^. Intratumoral CD8^+^ T_RM_ cells express inhibitory receptors, including CTLA-4, TIM-3, TIGIT, and CD39, which are largely confined to CD8^+^ T_RM_ cells^12–14^. Co-expression of CD39 and CD103 identifies tumor-reactive CD8^+^ T cells, strongly indicating a functional relevance for CD8^+^ T_RM_ cells in the control of lung tumors^14^. To date, it remains unknown whether tissue-resident lymphocytes other than CD8^+^ T_RM_ cells accumulate in human lung tumors and whether these cells share common traits with respect to expression of inhibitory receptors, chemokine receptors, and effector functions.

Here, we dissected the landscape of tissue-resident lymphocytes in patients with lung cancer in order to map common and distinct features of tissue-resident lymphocytes in human lung tumors. We show that trNK cells and CD8^+^ T_RM_ cells accumulated toward the center of non-small cell lung carcinomas (NSCLC), associated with slightly overlapping yet distinct chemokine receptor expression patterns on trNK cells and CD8^+^ T_RM_ cells. In line with low expression of immune checkpoint receptors, trNK cells from the tumor center were responsive to *ex vivo* target cell stimulation.

## Material and methods

### Lung tissue collection

Thirty-seven patients undergoing lobectomy for suspected lung cancer were included, with patient-matched tumor tissues (peritumoral, margin, center) from 17 patients, non-matched tumor-distal tissue from 19 patients, and matched tumor-distal tissue from one patient. All tumor tissues were derived from NSCLC, while two additional tumor-distal tissues were derived from carcinoid tumors. Definition of the distinct tumor areas was controlled by pathologists. Patients with records of preoperative chemotherapy and/or radiotherapy, strong immunosuppressive medication and/or hematological malignancy were excluded. Clinical and demographic details of the patients are summarized in Table 1. The Regional Ethical Review Board in Stockholm approved the study (permit 2018/1819–31/1), and all donors gave informed written consent prior to sample collection.

**Table 1.** Clinical and demographical details of the patients included in the study.

	Distal (*n* = 20)	Tumor (*n* = 17)
Female/male	11/9	10/7
Age (year), mean ± SD	71.7 ± 9.4	71 ± 8.8
Smoker	80% (16)	94% (17)
*Pathology*	*% (n)*	*% (n)*
Adenocarcinoma (ADC)	75% (15)	60% (10)
Squamous cell carcinoma (SCC)	10% (2)	40% (7)
Carcinoid	10% (2)	0% (0)
Other	5% (1)	0% (0)
*Tumor stage* IA IB IIA IIB IIIA	*% (n)* 45% (9) 20% (4) 0% (0) 10% (2) 15% (3)	*% (n)* 11.7% (2) 35.3% (6) 11.8% (2) 23.5% (4) 17.6% (3)

### Processing of tissue specimens

Lung tissue was processed as previously described^15^. Briefly, macroscopically tumor-free human lung tissue, taken either as distal as possible from the tumor site (defined as ‘distal’), or from a site in close proximity (within 2.5 cm) to the tumor (defined as ‘peritumoral’), together with a part taken from the tumor margin and tumor center, were transferred into ice-cold PBS or Krebs-Henseleit buffer^15^ and stored at 4°C for maximal 18 h until processing. Tissue was digested using collagenase II (Sigma-Aldrich) and DNase (Roche) for 30 min at 37°C. After digestion, RPMI supplemented with 10% FCS (Thermo Scientific), 1 mM L-glutamine (Invitrogen), 100 U/ml penicillin, and 50 μg/ml streptomycin (Invitrogen) (R10 medium) was added, and the cell suspension was passed through a 40-μm cell strainer and washed twice in R10 medium. Mononuclear cells from lung cell suspensions were isolated by density gradient centrifugation (Lymphoprep, Axis Shield).

### Flow cytometry

Cells were stained with antibodies against extracellular and intracellular proteins (Supplementary Table S1). Secondary stainings were performed using streptavidin BB630 (BD Biosciences), or streptavidin BV650 (Biolegend) and Live/Dead Aqua (Molecular probes, Life Technologies). After surface staining, cells were fixed and permeabilized using a FoxP3/Transcription Factor staining kit (eBioscience). Samples were analyzed on a BD Symphony equipped with five lasers (BD Biosciences), and data were analyzed using FlowJo 10.7.1 (BD Biosciences).

### Degranulation assay

NK cell degranulation was analyzed as previously described^15^. In brief, fresh lung mononuclear cells were resuspended in R10 medium and rested for 15–18 h at 37°C. Cells were cocultured with or without K562 cells for 6 h with anti-human CD107a (BV421, H4A3; BD Biosciences). Brefeldin A and monensin (GolgiPlug and GolgiStop, BD Biosciences) were added for the last 5 h of incubation.

### Statistics

Statistics were analyzed in GraphPad Prism 9. Wilcoxon matched-pairs signed rank test or Mann–Whitney test were used for matched or unmatched pairs of data, respectively, unless otherwise stated.

## Results

### Tissue-resident NK cells and CD8^+^ T_RM_ cells are enriched in human NSCLC tumors

**Figure 1.**
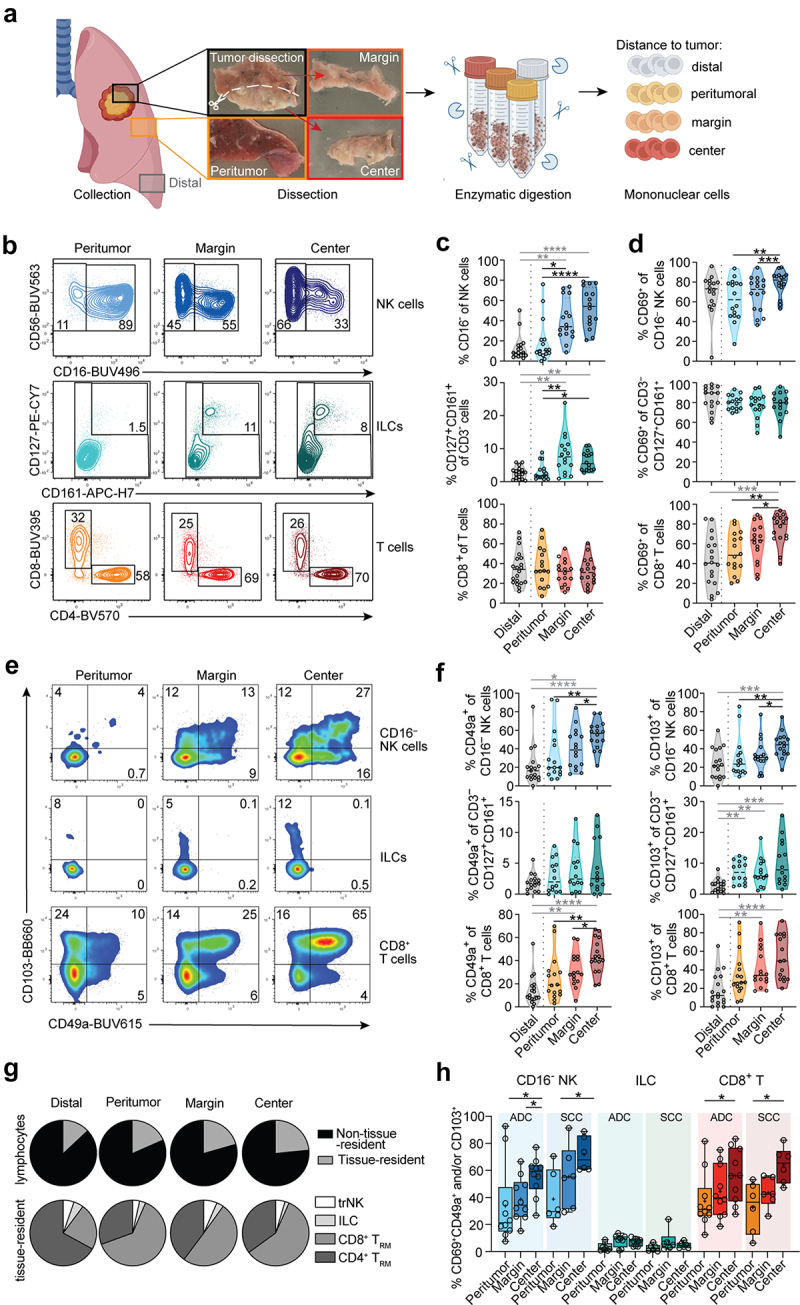
Distribution of CD16^−^ NK cells, ILCs, and CD8^+^ T cells in human lung tumors. (a) Graphical overview of workflow. Created with BioRender.com. (b) Representative contour plots and (c) summary of frequencies of CD16^−^ NK cells, CD127^+^CD161^+^CD3^−^ ILCs, and CD8^+^ T cells, respectively. (d) Frequencies of CD69^+^ cells among CD16^−^ NK cells, ILCs, and CD8^+^ T cells in tumor-free and intratumoral tissues (*n* = 16–20) are shown. (e) Representative pseudocolor plots and (f) frequencies of CD49a and CD103 expression on CD16^−^ NK cells, ILCs, and CD8^+^ T cells in tumor-free and tumor areas (*n* = 14–18). (g) Pie charts showing the composition of tissue-resident cells within the total lymphocyte (upper row) or tissue-resident (lower row) population (*n* = 15–17). (h) Frequencies of trNK cells, ILCs, and CD8^+^ T_RM_ cells co-expressing CD69, CD49a, and/or CD103 (*n* = 14–16). (c, d, f, h) Friedman test, Dunn’s multiple comparisons test (patient-matched, black); Kruskal–Wallis test (unmatched, gray). *p < 0.05, **p < 0.01, ***p < 0.001, ****p < 0.0001.

First, we analyzed the landscape of CD16^−^ NK cells, ILCs, and T cell subsets in tumor-free (distal), peritumoral, tumor margin, and tumor center tissues of human lung tumors (Figure 1a; gating strategies in Figure S1A-D). Frequencies of CD16^−^ NK cells of total NK cells as well as of ILCs (defined as CD3^−^CD127^+^CD161^+^ cells) of total CD3^−^ lymphocytes were higher toward the tumor center compared to peritumoral or distal tissue (Figure 1b, c). In contrast, frequencies of CD8^+^ and CD4^+^ T cells of total T cells were similar between the different tissue areas (Figure 1b, c, Figure S1B).

Expression of CD69, a marker for tissue-resident lymphocytes, was more frequently expressed toward the tumor center on CD16^−^ NK cells and CD8^+^ T cells and in a similar but non-significant trend on CD4^+^ T cells, while nearly all ILCs expressed CD69 at all locations studied (Figure 1d, Figure S1E, F). Next, we analyzed the distribution of cell subsets with additional features of tissue-residency, such as the expression of CD49a and CD103. Both CD16^−^ NK cells and CD8^+^ T cells expressed CD49a and CD103 more frequently in the tumor center as compared to the tumor margin, peritumoral, and distal sites (Figure 1e, f). In contrast, CD4^+^ T cells expressed similar levels of CD49a and CD103 throughout all areas (Figure S1E, F). On ILCs, CD49a expression was generally low; however, CD103 expression was elevated in the tumor center as compared to tumor-free distal tissue (Figure 1e, f). In line with these results, tissue-resident lymphocytes were slightly more frequent in the tumor center (Figure 1g). Among the tissue-resident lymphocytes, CD8^+^ T_RM_ cells comprised the predominant tissue-resident population in the tumor and peritumoral areas, while CD4^+^ T_RM_ cells dominated in tumor-distal tissue (Figure 1g, Figure S1G), indicating proportional changes of the lymphocyte composition in tumor-free tissue in close proximity to the tumor. The stratification of data based on the NSCLC-tumor histotype, i.e. adenocarcinoma (ADC) or squamous cell carcinoma (SCC), revealed comparable patterns of trNK cell and CD8^+^ T_RM_ cell accumulation toward the tumor center, independent of tumor type specificity or cancer stage (Figure 1h, Figure S1H-J). Together, our results suggest an accumulation of tissue-resident lymphocytes, mainly constituted by CD8^+^ T_RM_ cells, in the center of human NSCLC tumors.

### CXCR3^+^CXCR6^+^ trNK cells and CD8^+^ T_RM_ cells accumulate within the tumor center

**Figure 2.**
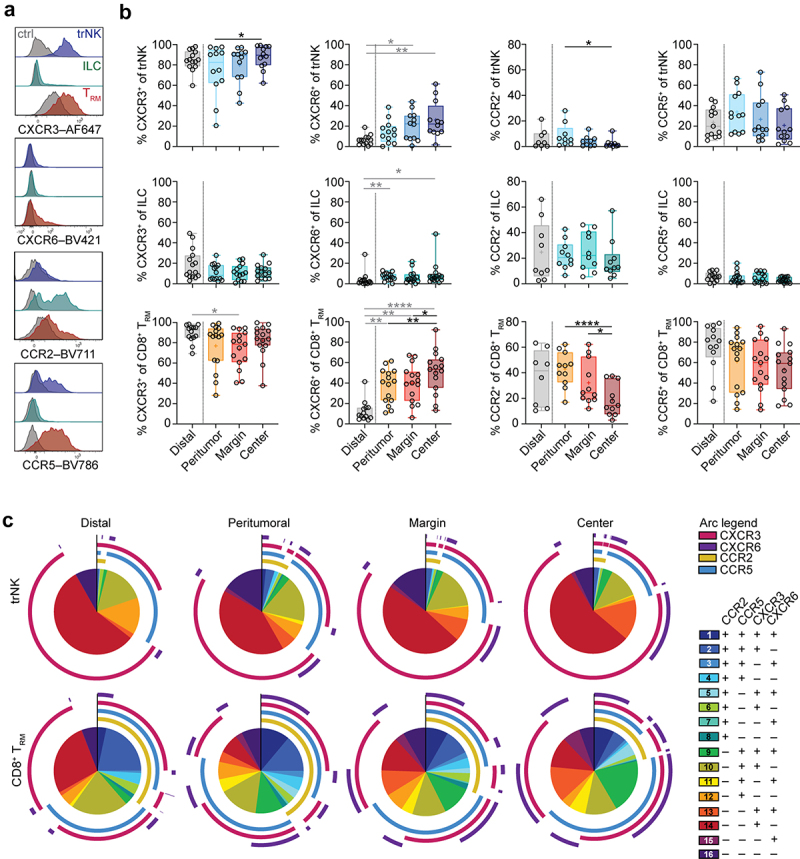
Chemokine receptor expression on tissue-resident lymphocyte subsets in lung tumors. (a) Representative overlays of expression of CXCR3, CXCR6, CCR2, and CCR5 on trNK cells (blue), ILCs (green), and CD8^+^ T_RM_ cells (red) from lung tumor center. Gray histograms represent FMO controls. (b) Frequencies of trNK cells, ILCs, and CD8^+^ T_RM_ cells expressing CXCR3, CXCR6, CCR2, or CCR5 in tumor-free tissue or tumor tissues (*n* = 8–16). Friedman test, Dunn’s multiple comparisons test (patient-matched, black); Kruskal–Wallis test (unmatched, gray). Mean indicated as ‘+’. *p < 0.05, **p < 0.01, ****p < 0.0001. (c) SPICE analysis of co-expression patterns of CXCR3, CXCR6, CCR2, and CCR5 on trNK cells, ILCs, and CD8^+^ T_RM_ cells in different tumor-free tissues and in tumor tissues (*n* = 6–8).

The increase in trNK cells, ILCs, and CD8^+^ T_RM_ cells toward the tumor center suggested a selective recruitment or retention of these cells at this location. To identify whether tissue-resident lymphocyte subsets share common tumor-infiltration capacities, we analyzed the expression of chemokine receptors relevant for mediating recruitment of leukocytes to the tumor microenvironment^16–18^. Expression of CXCR3, CXCR6, CCR2, and CCR5 differed between trNK cells, ILCs, CD8^+^ T_RM_ cells, and CD4^+^ T_RM_ cells (Figure 2a, Figure S2A), as well as between locations within the tumor and tumor-distal lung tissue (Figure 2b). CXCR3 expression was high on trNK cells and CD8^+^ T_RM_ cells (Figure 2b, c) and did not differ between ADC and SCC (Figure S2B, C). Notably, the frequency of CXCR6^+^ trNK cells and CD8^+^ T_RM_ cells increased toward the tumor center (Figure 2b, c). However, this observation was specific to SCC and not observed in ADC (Figure S2B, C). Conversely, the proportion of trNK cells and CD8^+^ T_RM_ cells expressing CCR2 decreased (Figure 2b, c). This reduction was particularly noticeable on trNK cells derived from SCC, potentially attributable to their overall low expression. Nonetheless, it was evident in both cancer subtypes for CD8^+^ T_RM_ cells (Figure S2B, C). CCR5 expression on trNK cells and CD8^+^ T_RM_ cells was similar at all locations (Figure 2b, c). Except for CCR2, chemokine receptor expression was low on ILCs, with no or only minor changes throughout all areas (Figure 2b, Figure S2B, C). Co-expression patterns of chemokine receptors were considerably more diverse on CD8^+^ T_RM_ cells than on trNK cells (Figure 2c). Strong increases toward the tumor center were observed for CXCR3^+^CXCR6^+^ cells (population 13) and CCR5^+^CXCR3^+^CXCR6^+^ CD8^+^ TRM cells (population 9), concurrent with a decrease in CCR5 single-positive (population 12) as well as CCR2^+^CCR5^+^CXCR6^+^ (population 3) CD8^+^ T_RM_ cells (Figure 2c). A large proportion of CXCR3^+^CXCR6^+^ CD8^+^ T_RM_ cells but not trNK cells also co-expressed CCR5 (population 9), indicating a potential role for CCR5 specifically on CD8^+^ T_RM_ cells. In contrast, CD8^+^ T_RM_ cells expressing only CXCR3 (population 14) and co-expressing CXCR3, CCR2, and CCR5 (population 2) were largely confined to the distal non-tumor area (Figure 2c).

Altogether, although chemokine receptor co-expression patterns differed between trNK cells and CD8^+^ T_RM_ cells, increased CXCR6 expression on both cell types toward the tumor center indicate that CXCR6 constitutes a key-receptor for mediating the accumulation of trNK cells and CD8^+^ T_RM_ cells in lung tumors.

### Immune checkpoint receptor expression increases on tumor-infiltrating *CD8^+^* T_RM_ cells but not trNK cells or ILCs

**Figure 3.**
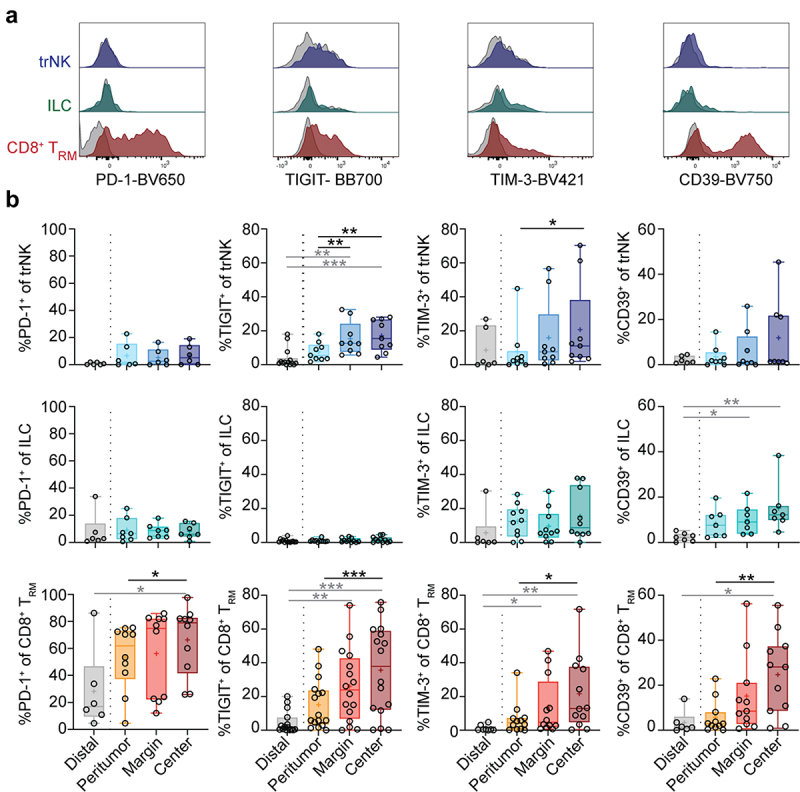
Immune checkpoint receptor expression on tissue-resident lymphocytes in lung tumors. (a) Representative overlays of expression of PD-1, TIGIT, TIM-3, and CD39 on trNK cells, ILCs, and CD8^+^ T_RM_ cells in the tumor center. Gray histograms represent FMO controls. (b) Frequencies of trNK cells, ILCs, and CD8^+^ T_RM_ cells expressing PD-1, TIGIT, TIM-3, or CD39 in different tumor-free tissues or tumor tissues (*n* = 6–16). Friedman test, Dunn’s multiple comparisons test (patient-matched, black); Kruskal–Wallis test (unmatched, gray). Mean indicated as ‘+’. *p < 0.05, **p < 0.01, ***p < 0.001.

Despite the capacity to infiltrate tumors, intratumoral NK cells and T cells have been suggested to be largely functionally inert^19,20^, in part resulting from upregulation of immune checkpoint receptors/exhaustion markers such as PD-1^21,22^, TIM-3^23,24^, TIGIT^25^, and CD39^26,27^. Since little is known about the expression of immune checkpoint receptors on tissue-resident innate lymphocytes, we assessed their expression on trNK cells, ILCs, CD8^+^ T_RM_, and CD4^+^ T_RM_ cells in the distinct tumor locations (Figure 3a, b and Figure S3A). In most donors, immune checkpoint receptors were expressed by few trNK cells and ILCs at all locations, whereas CD8^+^ T_RM_ cells displayed high immune checkpoint receptor expression in the tumor tissues (Figure 3a, b). Expression of TIGIT and TIM-3 on trNK cells, and all immune checkpoint receptors analyzed on CD8^+^ T_RM_ and CD4^+^ T_RM_ cells increased toward the tumor center (Figure 3b, Figure S3A). Although TIGIT was expressed by a higher frequency of trNK cells in the tumor center compared to peritumoral and distal tissue, expression remained considerably lower than that of CD8^+^ T_RM_ cells at all locations (Figure 3b). In contrast to other immune checkpoint receptors, TIGIT was not expressed on ILCs (Figure 3b), which, however, more frequently expressed CD39 toward the tumor center (Figure 3b). Immune checkpoint receptor expression on trNK cells, ILCs, and CD8^+^ T_RM_ cells did not differ significantly between ADC and SCC (Figure S3B).

Together, our data indicate that CD8^+^ T_RM_ cells in lung tumors would be substantially more affected by common immune checkpoint therapies than trNK cells and ILCs.

### Different activation profiles of tumor-infiltrating tissue-resident lymphocytes

**Figure 4.**
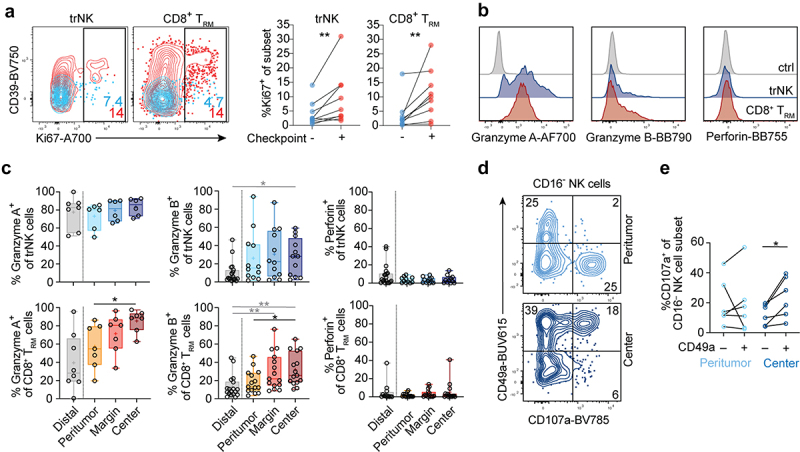
Cytotoxic profiles of trNK cells and CD8^+^ T_RM_ cells in lung tumors. (a) Representative contour plots (left) and summary data (right) of Ki67 expression on checkpoint receptor negative (blue) or positive (red) trNK and CD8^+^ T_RM_ cells (*n* = 9). (b) Representative overlays (tumor center) and (c) summary of data showing frequencies of granzyme A, granzyme B, and perforin expression in trNK cells and CD8^+^ T_RM_ cells in different tumor and tumor-free tissue areas (*n* = 7–19). (d) Representative contour plots displaying degranulation in response to K562 target cells. CD16^−^ NK cells from peritumoral tissue or tumor center were gated. (e) Frequencies of CD107a^+^CD49a^−^CD16^−^ and CD107a^+^CD49a^+^CD16^−^ NK cells following stimulation with K562 target cells. Data from unstimulated controls have been subtracted (*n* = 6). (c) Friedman test, Dunn’s multiple comparisons test (patient-matched, black); Kruskal–Wallis test (unmatched, gray). (a, e) Wilcoxon test. *p < 0.05, **p < 0.01.

Low expression of immune checkpoint receptors on trNK cells led us to assess functional properties in comparison to CD8^+^ T_RM_ cells (Figure 4). Immune checkpoint receptor-expressing trNK cells and CD8^+^ T_RM_ cells expressed more Ki67 compared to checkpoint receptor-negative cells (Figure 4a). Furthermore, the tumor center was enriched with trNK cells and CD8^+^ T_RM_ cells expressing granzymes (Figure 4b, c). While granzyme A expression did not differ between ADC and SCC, granzyme B expression was significantly higher on CD8^+^ T_RM_ cells in SCC in the tumor center compared to the corresponding region in ADC (Figure S4A, B). However, both trNK cells and CD8^+^ T_RM_ cells lacked perforin expression (Figure 4b, c), which was consistent for both ADC and SCC (Figure S4A, B), despite it being readily detectable in CD16^+^ NK cells in all tumor areas (Figure S4C, D). As expected, ILCs and CD4^+^ T_RM_ cells did not express any or very low levels of cytotoxic effector molecules (Figure S4A, B). In contrast to trNK cells and CD8^+^ T_RM_ cells, their non-tissue-resident counterparts (defined as CD49a^−^CD103^−^CD16^−^ NK cells and CD49a^−^CD103^−^CD8^+^ T cells, respectively) expressed granzyme A and B less frequently in the tumor center (Figure S4C, D). These expression patterns suggest different cytotoxic capacities of tissue-resident and non-tissue-resident lymphocyte subsets toward the center of human lung tumors. Indeed, the analysis of target cell responsiveness revealed that CD49a^+^CD16^−^ NK cells from the tumor center degranulated stronger than CD49a^−^CD16^−^ NK cells (Figure 4d, e), suggesting the functional competence of trNK cells in the tumor center.

Together, these results reveal different effector profiles of tissue-resident lymphocyte subsets depending on their location within lung tumors. While conventional intratumoral NK cell and T cell subsets are considered hypofunctional^19,20^, our results suggest that trNK cells are tumor target cell-responsive.

## Discussion

Here, we studied the distribution and phenotypic traits of tissue-resident lymphocyte subsets across human lung tumors and in tumor-free lung tissue.

Human trNK cells have been described in tumor-free lung^2^, however, their distribution in lung tumors is largely unexplored. Our study revealed an accumulation of trNK cells, ILCs, and CD8^+^ T_RM_ cells toward the center of human lung tumors, which may, in particular for trNK cells and CD8^+^ T_RM_ cells, be linked to chemokine receptors such as CXCR3 and CXCR6, which are relevant for NK cell and T cell infiltration into tumors^28–30^. Both CXCR3 and CXCR6 were expressed at highest frequencies on tumor center trNK cells and CD8^+^ T_RM_ cells, respectively. Additionally, our data suggest that the CCL5/CCR5 axis might play a role in tumor-infiltration of CD8^+^ T_RM_ cells, in line with a strong correlation between the expression of CCL5 and CD8^+^ T cell infiltration across different types of cancer^31^. In addition to varying infiltration capacities, the observed phenotype could have been induced by the tumor-microenvironment or in response to other infiltrating leukocytes. For example, circulating NK cells co-cultured with a head and neck SCC cell line and IL-15 upregulated CD49a and CD103, indicating promotion of a tissue-residency phenotype via the tumor microenvironment^32^. Therefore, other mechanisms leading to the accumulation of specific cell subsets such as apoptosis, metabolic differences, and lineage-dependent development must be considered.

Despite functional exhaustion, upregulation of immune checkpoint receptors on CD8^+^ T_RM_ cells and trNK cells, albeit at low frequencies, indicates an immune-activating environment^33^. Indeed, tumor-infiltrating CD103^+^ T_RM_ cells are more functional and cytotoxic as compared to their CD103^−^ counterparts (reviewed in^34^). Furthermore, the expression of both granzyme A and granzyme B was highest in trNK cells and CD8^+^ T_RM_ cells in the tumor center. Notably, granzyme B expression was significantly higher on CD8^+^ T_RM_ cells in the tumor center in SCC compared to ADC.

Intratumoral CD49a^+^ NK cells were readily responsive to tumor target cells, in contrast to previous reports^35^. However, effector cell function in the tumor might be compromised due to lack of perforin expression in these cell subsets. It remains to be determined whether trNK cells can rapidly upregulate perforin upon stimulation as shown for CD8^+^ T_RM_ cells in influenza infection^1^. Together with a low immune checkpoint receptor expression, this would make trNK cells a relevant alternative for future therapeutic applications in solid tumors. Manipulating the interaction of intratumoral chemokines with their cognate receptors on trNK cells and CD8^+^ T_RM_ cells might represent another suitable immunotherapeutic approach to modulate cell subset infiltration and retention (reviewed in^36^). Additionally, trNK and/or CD8^+^ T_RM_ cells could be expanded and primed *ex vivo* and harnessed for targeted lysis of the tumor cells. Future analyses will determine whether trNK cells and CD8^+^ T_RM_ cells can be directly or indirectly harnessed for optimization and development of novel treatment strategies for patients with solid tumors.

## Supplementary Material

Supplemental Material

## Data Availability

The data that support the findings of this study are available from the corresponding author, NM, upon reasonable request.
