# Supplementary material for: Accumulation of tissue-resident natural killer cells, innate lymphoid cells, and CD8+ T cells towards the center of human lung tumors
Source: Oncoimmunology. 2023 Jul 11;12(1):2233402. doi: 10.1080/2162402X.2023.2233402 (PMC10337494; doi:10.1080/2162402X.2023.2233402)
Supplement: Supplemental Material [file KONI_A_2233402_SM1221.zip › Supplementary Table 1.pdf]

Supplementary Table 1

Target	Clone	Fluorochrome	Company
Extracellular			
CD3	UCHT1	PE/Cy5	Beckman Coulter
CD4	RPAT4	BV570	Biolegend
CD4	RPAT4	BB630	BD Biosciences
CD8	RPA-T8	BB700	BD Biosciences
CD8	RPA-T8	BUV737	BD Biosciences
CD8	RPA-T8	BUV395	BD Biosciences
CD14	MφP9	Horizon V500	BD Biosciences
CD16	3G8	BUV496	BD Biosciences
CD19	HIB19	Horizon V500	BD Biosciences
CD45	HI30	BUV805	BD Biosciences
CD49a	SR84	BUV615	BD Biosciences
CD56	NCAM16.2	BUV563	BD Biosciences
CD57	QA17A04	BUV605	Biolegend
CD103	Ber-ACT8	BB660	BD Biosciences
CD69	FN50	BV650	Biolegend
CD69	FN50	PE-CF594	BD Biosciences
CD69	FN50	BV750	BD Biosciences
CD39	TU66	BV750	BD Biosciences
CD127	A019D5	BV711	Biolegend
CD161	HP3-3G10	BV605	Biolegend
CD161	HP3-3G10	APC-Fire 750	Biolegend
CXCR3	G025H7	Alexa Fluor 647	Biolegend
CXCR6	K041E5	BV421	Biolegend
CCR2	K036C2	BV711	Biolegend
CCR5	J418F1	BV650	Biolegend
CCR5	J418F1	BV785	Biolegend
KIR2DL2/S2/L3	GL183	PE/Cy5.5	Beckman Coulter
NKG2A	Z1991.10	PE	Beckman Coulter
NKG2A	REA110	VioBrightFITC	Miltenyi
PD-1	EH12.1	BUV737	BD Biosciences
PD-1	REA1165	biotin	Miltenyi
TIGIT	741182	BB700	BD Biosciences
TIM-3	7D3	BV711	BD Biosciences
TIM-3	F38-2E2	BV421	Biolegend
Intracellular			
Granzyme A	CB9	Alexa Fluor 700	Biolegend
Ki67	B56	Alexa Fluor 700	BD Biosciences
perforin	dG9	BB755	BD Biosciences
Granzyme B	GB11	BB790	BD Biosciences
