# Supplementary figures and images for: Accumulation of tissue-resident natural killer cells, innate lymphoid cells, and CD8+ T cells towards the center of human lung tumors
Source: Oncoimmunology. 2023 Jul 11;12(1):2233402. doi: 10.1080/2162402X.2023.2233402 (PMC10337494; doi:10.1080/2162402X.2023.2233402)

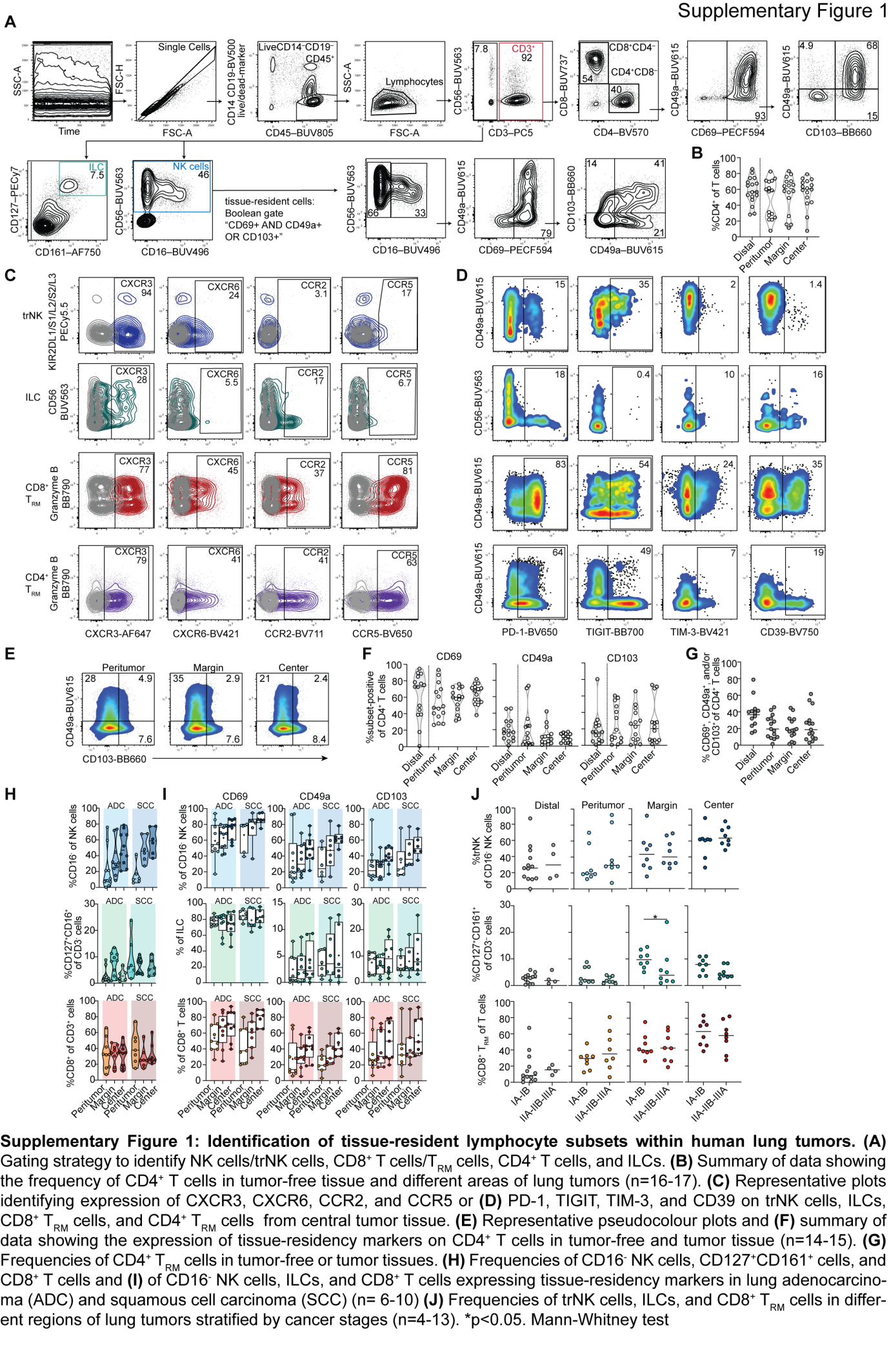


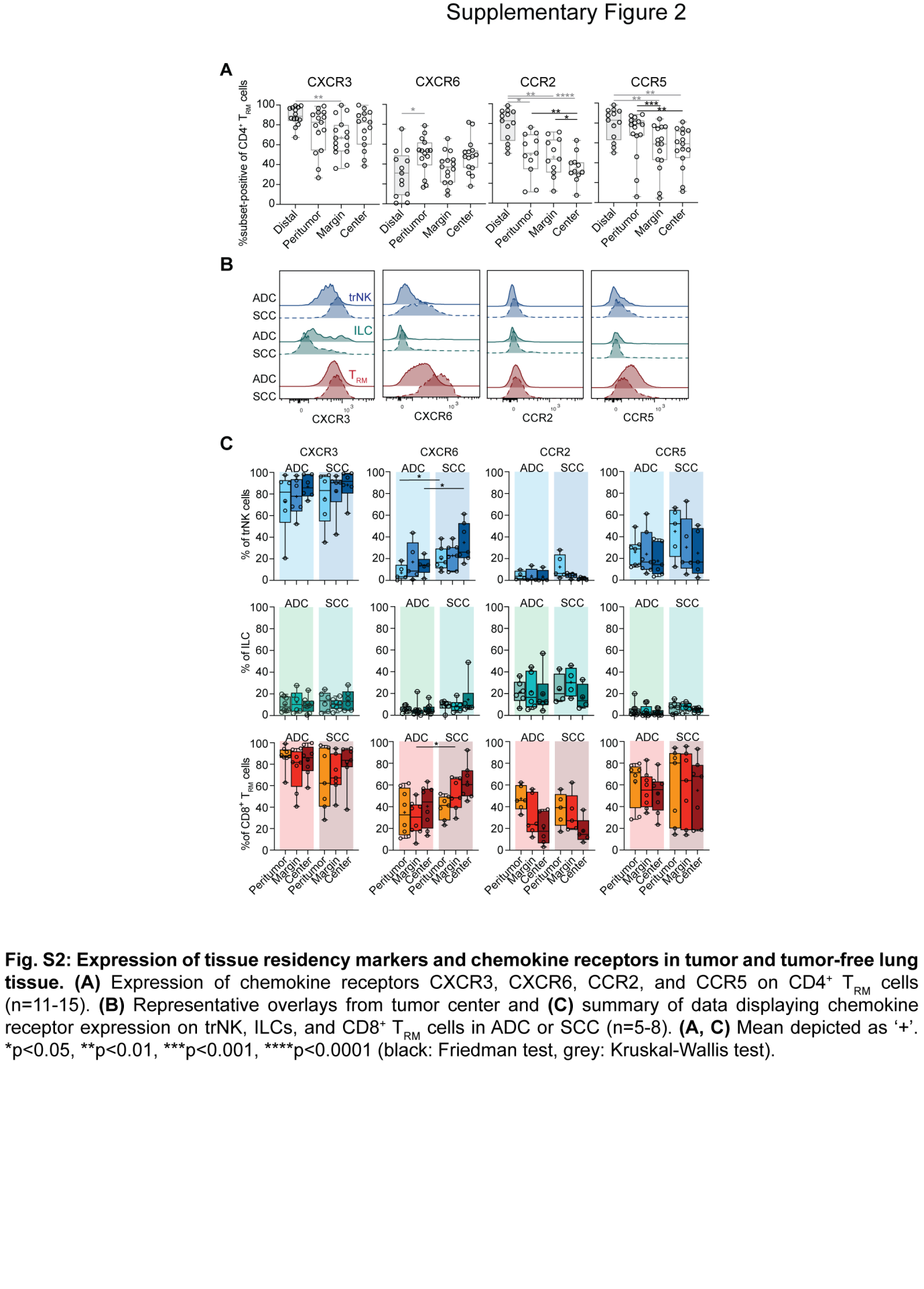


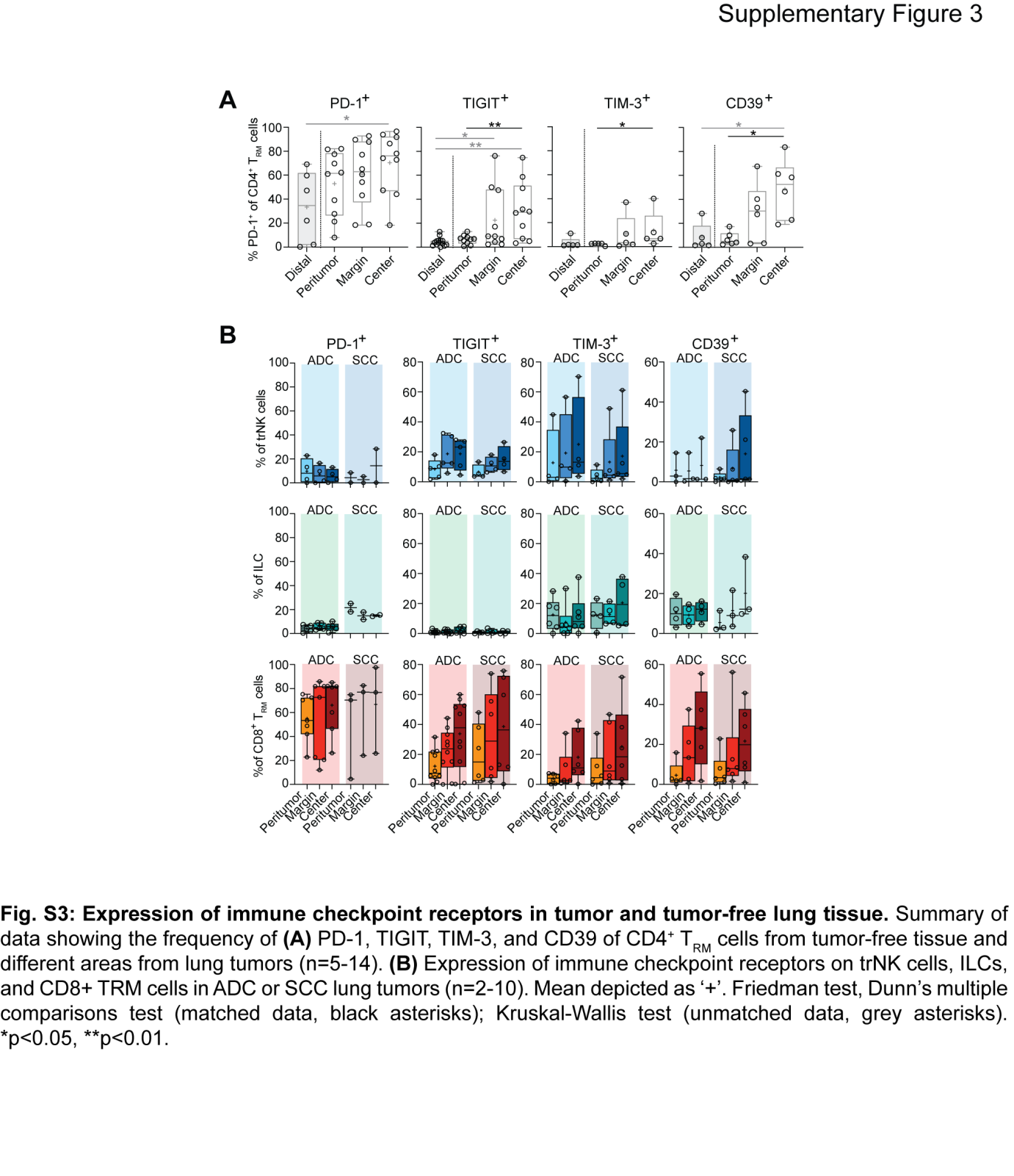

Supplement: Supplemental Material [file KONI_A_2233402_SM1221.zip › Figure S1, S2 and S3.docx]
